# Targeted Sequencing Identifies the Genetic Variants Associated with High-altitude Polycythemia in the Tibetan Population

**DOI:** 10.1007/s12288-021-01474-1

**Published:** 2021-08-03

**Authors:** Zhiying Zhang, Lifeng Ma, Xiaowei Fan, Kun Wang, Lijun Liu, Yiduo Zhao, ZhiPeng Zhao, Han Zhang, Tian Liang, Wenxue Dong, Peng Cai, Yansong Li, Jing Li, Songhua Zhou, Longli Kang

**Affiliations:** 1 Key Laboratory for Molecular Genetic Mechanisms and Intervention Research on High Altitude Disease of Tibet Autonomous Region, Xianyang, China; 2 Key Laboratory of High Altitude Environment and Genes Related to Diseases of Tibet Autonomous Region, School of Medicine, Xizang Minzu University, Xianyang, 712082 Shaanxi China; 3 Ministry of Education Key Laboratory of Contemporary Anthropology, Collaborative Innovation Center for Genetics and Development, School of Life Sciences, Fudan University, Shanghai, 200433 China; 4 Foshan University, Foshan, 528000 Guangdong China

**Keywords:** High-altitude polycythemia, Highland people, SNPs

## Abstract

High-altitude polycythemia (HAPC) is characterized by excessive proliferation of erythrocytes, resulting from the hypobaric hypoxia condition in high altitude. The genetic variants and molecular mechanisms of HAPC remain unclear in highlanders. We recruited 141 Tibetan dwellers, including 70 HAPC patients and 71 healthy controls, to detect the possible genetic variants associated with the disease; and performed targeted sequencing on 529 genes associated with the oxygen metabolism and erythrocyte regulation, utilized unconditional logistic regression analysis and GO (gene ontology) analysis to investigate the genetic variations of HAPC. We identified 12 single nucleotide variants, harbored in 12 genes, associated with the risk of HAPC (4.7 ≤ odd ratios ≤ 13.6; 7.6E − 08 ≤ *p*-value ≤ 1E − 04). The pathway enrichment study of these genes indicated the three pathways, the PI3K-AKT pathway, JAK-STAT pathway, and HIF-1 pathway, are essential, which *p*-values as 3.70E − 08, 1.28 E − 07, and 3.98 E − 06, respectively. We are hopeful that our results will provide a reference for the etiology research of HAPC. However, additional genetic risk factors and functional investigations are necessary to confirm our results further.

**Supplementary Information:**

The online version contains supplementary material available at 10.1007/s12288-021-01474-1.

## Introduction

The Qinghai-Tibet Plateau, namely the "roof of the world," is a high-altitude area with an elevation between 3000 and 5000 m. Hypoxia is one of the most critical characteristics of the high-altitude environment. High-altitude polycythemia (HAPC) is one of the chronic high-altitude diseases developed in Tibetan dwellers, characterized by an excessive number of circulating erythrocytes. The clinical diagnosis of HAPC requires a hemoglobin concentration (Hb) no less than 19 g/dL for females and 21 g/dL for males, respectively [1]. HAPC is often commonly accompanied by the symptoms, headache, dizziness, dyspnea, sleep disorders, or venous dilatation [1]. The incidence of HAPC in the Tibetan Plateau ranges from 5 to 18%, increasing with altitude, a severe public health problem in China and other Andean countries [2].

The compensation for prolonged hypobaric hypoxia is the main reason for the change of hemoglobin concentration through elevating oxygen-carrying, transportation, and exchange [3]. Elevated hemoglobin concentrations are crucial for adapting to high altitude environment. However, the pathological increased red blood cell may trigger blood viscosity, potentially slowing blood flow and inciting hypercoagulation, thrombosis, or tissue hypoxia, even further aggravate various circulatory, respiratory, digestive, and neurological diseases [4–6]. While the etiology and the pathophysiological of HAPC remain unclear, the incidence of HAPC showed significantly individual differences.

Recent studies revealed that HAPC in Tibetans or Han is associated with several gene polymorphisms, such as phosphatidylinositol-4,5-bisphosphonate 3-kinase, catalytic subunit delta gene (*PIK3CD*), collagen type IV α3 chain gene (*COL4A3*), integrin subunit alpha six gene (*ITGA6*), erb-b2 receptor tyrosine kinase four gene (*ERBB4*), EPH receptor A2 gene (*EPHA2*), angiotensinogen gene (*AGT*), and endothelial PAS domain protein one gene (*EPAS1*) [7–11] EPHA2 can disturb erythropoiesis by regulating EPO production, and EPAS1 contributes to genetic adaptation to high altitudes and the particular trait of the low Hb concentrations, conditions frequently observed among Tibetans [9, 12]. HAPC was found to lead to morphological changes and pathological damage to the gastric mucosa of patients. In particular, the kallikrein gene cluster (KLK1, KLK3, KLK7, KLK8, and KLK12) was upregulated > 17-fold. The elevated levels of KLK1, KLK3, KLK7, KLK8 and KLK12 may be closely associated with the hypertension, inflammation, obesity and other gastric injuries associated with polycythemia. Thus, the kallikreins are likely to potentially impact the development of HAPC [13]. Wang et al. [14] found that 19 serum proteins expressed differentially between patients and healthy controls with HAPC. Among them, C4A(The complement component 4A), C6(complement C6), CALR(Calreticulin), MASP1(Mannan Binding Lectin Serine Peptidase 1), and CNDP1(serum carnosinase) may enable researchers to use them as candidate plasma biomarkers for HAPC on account of their latent diagnostic, preventive and therapeutic values [14]. These results suggest that HAPC presents noticeable racial and significant individual differences in susceptibility.

In this study, we conducted a case–control study to investigate the association of genetic variants and the susceptibility of HAPC in the Tibetan population. We selected a group of genes considered involved in erythropoiesis and oxygen transportation by previous studies and using the target exome sequencing strategy to detect the coding variants. This work's overarching goal was to investigate associations between HAPC susceptibility and candidate genes that control oxygen metabolism in erythrocytes.

## Experimental Procedures

### Study Populations

HAPC was defined as males and females with Hb ≥ 21 g/dL and Hb ≥ 19 g/dL, respectively. We excluded individuals with chronic pulmonary diseases, lung cancer, or secondary polycythemia due to hypoxemia caused by certain chronic diseases. A total of 70 HAPC cases and 71 healthy controls of Tibetan inhabitants who lived in plateau areas above 2500 m were recruited from the Second People's Hospital of Tibet Autonomous Region. The cases include 35 males and 35 females. Control samples consisted of 39 males and 32 females. The age range of all samples was 20–60 years old. The Ethics Committee approved the study of the Xizang Minzu University with Ethics Number 201801.

### Epidemiological and Clinical Data

Participants' information was collected through physicians or from medical chart review. All study participants provided written, informed consent for participation. Also, we obtained 5 ml of peripheral blood specimens from each participant. All persons gave their informed consent before their inclusion in the study.

### Exome Sequencing and Bioinformatics of Target Genes

Targeted exome sequencing (agilent All exon kit V5 50 M) of 529 selected genes was conducted on all samples—these 529 genes with erythropoiesis and oxygen transportation by previous studies and database IPA (Ingenuity Pathway Analysis) and NCBI(National Center for Biotechnology Information), etc. [15]. Genomic DNA was extracted from the whole blood or saliva of individual subjects according to standard protocols. Double-stranded DNA was indexed and multiplexed in groups of six per lane and sequenced in 101-bp paired-end mode using the Illumina HiSeq 2000 platform. The mean on-target depth of each sample was 200X. The raw FASTQ files from the Illumina HiSeq were aligned to the human reference genome build 19 (GRCh37), and variants were called using SAMtools v0.1.18 and GATK v3.8. Low-quality variants were filtered as described previously [16]. Under the theory of "Common variants, common disease", we included the variants with minor allele frequency (MAF) > 0.05 in the Asian population HapMap database into the subsequent analysis.

### Statistical Analysis

The genotype frequencies of each SNP in the control subjects were checked using the Hardy–Weinberg equilibrium (HWE). HWE values > 0.01 were further analyzed. Genes *FER* (*ferritin*), *CREB5(*also called *CREBPA*, CRE (cyclic AMP response element)-binding proteins*)*, and *TP53* with HWE values < 0.01 in the control group, failing to conform to the HWE rule, were excluded from the study.

The effects of polymorphisms on the risk of HAPC were expressed as odds ratios (ORs) with 95% confidence intervals (95% CIs), evaluated by three genetic models (dominant, recessive, and additive models) using unconditional logistic regression analysis. The Multiple comparisons were corrected by FDR and Bonferroni method. FDR and Bonferroni-corrected *p* < 0.05 indicated a significant between-group difference. KEGG (Kyoto Encyclopedia of Genes and Genomes)-based enrichment analysis was conducted in R software [17]. All data were analyzed by R software package GenABEL20.

## Results

**Fig. 1:**
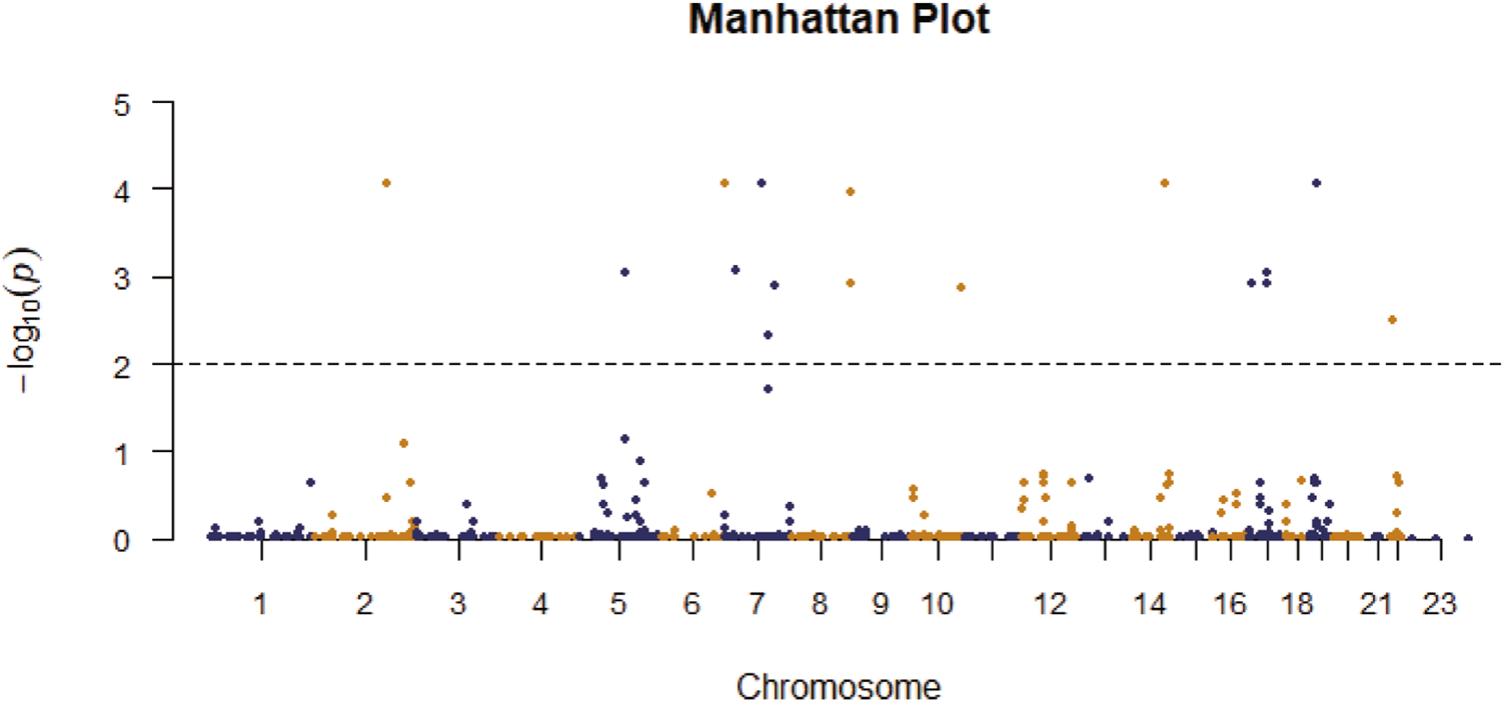
Manhattan plot of the p-value of the correlation between HAPC and SNP determined by false discovery rate (FDR) calculation

Table 1 displays the demographics of patients with HAPC and controls. Table 2 and Fig. 1 show the significantly differentiated genes, using the unconditional logistic regression analysis, false discovery rate (FDR) calculation, and Bonferroni correction. We observed significant discrepancies between the HAPC case and control groups in the dominant and additive genetic models. The results indicated the 12 genes, *PDK1*, *RUNDC3B*, *EPO*, *MET*, *PTK2*, *RELN*, *TDRD1*, *TCL1A*, *STAT3*, *STAT5A*, *IL12RB1*, and *NF2*, were associated with HAPC.

**Table 1:** Demographics of healthy controls and patients with high-altitude polycythemia

Variables	Tibetan	
	Case (n = 70)	Control (n = 71)
Male	35	39
Female	35	32

**Table 2:** Basic information on significant gene differences in highland people

SNP	Gene	CHR	Model	Alleles	Case (N)			Case	HWE	Control (N)			Control
				A/B	AA	AB	BB	MAF	Case	AA	AB	BB	MAF
rs529091195	PDK1	2	Dominant	/T	0	48	20	0.3529	1.87E − 06	0	17	54	0.1197
rs529091195	PDK1	2	Additive	/T	0	48	20	0.3529	1.87E − 06	0	17	54	0.1197
rs376837075	FER	5	Dominant	/T	0	66	2	0.485	4.13E − 17	0	33	35	0.243
rs376837075	FER	5	Additive	/T	0	66	2	0.485	4.13E − 17	0	33	35	0.243
rs527802276	RUNDC3B	7	Dominant	/A	0	61	7	0.449	8.42E − 13	0	29	41	0.2071
rs527802276	RUNDC3B	7	Additive	/A	0	61	7	0.449	8.42E − 13	0	29	41	0.2071
rs773485910	EPO	7	Dominant	T/-	0	46	22	0.338	6.99E − 06	0	22	49	0.155
rs773485910	EPO	7	Additive	T/-	0	46	22	0.338	6.99E − 06	0	22	49	0.155
rs397889442	RELN	7	Dominant	A/-	0	41	25	0.311	1.11E − 04	0	18	47	0.139
rs397889442	RELN	7	Additive	A/-	0	41	25	0.311	1.11E − 04	0	18	47	0.139
rs548208912	CREB5	7	Dominant	T/-	0	61	3	0.477	8.11E − 15	0	33	34	0.246
rs548208912	CREB5	7	Additive	T/-	0	61	3	0.477	8.11E − 15	0	33	34	0.246
rs551879100	MET	7	Dominant	T/-	0	58	10	0.427	6.20E − 11	0	33	38	0.232
rs551879100	MET	7	Additive	T/-	0	58	10	0.427	6.20E − 11	0	33	38	0.232
rs372806706	PTK2	8	Dominant	A/-	0	61	6	0.455	3.86E − 13	0	31	39	0.221
rs372806706	PTK2	8	Additive	A/-	0	61	6	0.455	3.86E − 13	0	31	39	0.221
rs369382658	PTK2	8	Dominant	A/-	0	61	6	0.455	3.86E − 13	0	31	39	0.221
rs369382658	PTK2	8	Additive	A/-	0	61	6	0.455	3.86E − 13	0	31	39	0.221
rs11285127	TDRD1	10	Dominant	A/-	0	49	19	0.36	7.34E − 07	0	21	45	0.159
rs11285127	TDRD1	10	Additive	A/-	0	49	19	0.36	7.34E − 07	0	21	45	0.159
rs142205645	TCL1A	14	Dominant	/AG	0	50	18	0.368	5.55E − 07	0	19	52	0.134
rs142205645	TCL1A	14	Additive	/AG	0	50	18	0.368	5.55E − 07	0	19	52	0.134
rs141204613	TP53	17	Dominant	/TTT	1	64	3	0.485	1.49E − 14	0	35	31	0.265
rs141204613	TP53	17	Additive	/TTT	1	64	3	0.485	1.49E − 14	0	35	31	0.265
rs558351915	STAT3	17	Dominant	A/-	0	55	11	0.417	6.74E − 10	0	27	38	0.208
rs558351915	STAT3	17	Additive	A/-	0	55	11	0.417	6.74E − 10	0	27	38	0.208
rs779456792	STAT5A	17	Dominant	T/-	0	52	16	0.382	5.58E − 08	0	26	45	0.183
rs779456792	STAT5A	17	Additive	T/-	0	52	16	0.382	5.58E − 08	0	26	45	0.183
rs548702753	IL12RB1	19	Dominant	/T	2	47	16	0.392	2.66E − 05	0	18	48	0.136
rs548702753	IL12RB1	19	Additive	/T	2	47	16	0.392	2.66E − 05	0	18	48	0.136
rs769771815	NF2	22	Dominant	T/-	0	48	18	0.364	7.44E − 07	0	23	44	0.171
rs769771815	NF2	22	Additive	T/-	0	48	18	0.364	7.44E − 07	0	23	44	0.171

SNP	Gene	CHR	Model	HWE	OR	95%CI		P	P1	P2			
				Control		L95	U95						
rs529091195	PDK1	2	Dominant	0.583	7.624	3.585	16.21	1.31E − 07	8.74E − 05	0.0004026			
rs529091195	PDK1	2	Additive	0.583	7.624	3.585	16.21	1.31E − 07	8.75E − 05	0.0004031			
rs376837075	FER	5	Dominant	0.007	35	7.928	15.45	2.70E − 06	0.0009179	0.008261			
rs376837075	FER	5	Additive	0.007	35	7.928	15.45	2.70E − 06	0.000919	0.008272			
rs527802276	RUNDC3B	7	Dominant	0.0312	12.32	4.933	30.77	7.58E − 08	0.0000874	0.000232			
rs527802276	RUNDC3B	7	Additive	0.0312	12.32	4.933	30.77	7.58E − 08	0.0000875	0.0002323			
rs773485910	EPO	7	Dominant	0.197	4.657	2.278	9.519	2.47E − 05	0.004731	0.0757			
rs773485910	EPO	7	Additive	0.197	4.657	2.278	9.519	2.47E − 05	0.004737	0.0758			
rs397889442	RELN	7	Dominant	0.593	4.282	2.05	8.944	0.0001087	0.01958	0.3328			
rs397889442	RELN	7	Additive	0.593	4.282	2.05	8.944	0.0001087	0.0196	0.3333			
rs548208912	CREB5	7	Dominant	0.007	20.95	5.977	73.42	1.99E − 06	0.0008717	0.006102			
rs548208912	CREB5	7	Additive	0.007	20.95	5.977	73.42	1.99E − 06	0.0008729	0.00611			
rs551879100	MET	7	Dominant	0.015	6.679	2.95	15.12	5.26E − 06	0.001239	0.01611			
rs551879100	MET	7	Additive	0.015	6.679	2.95	15.12	5.26E − 06	0.001241	0.01613			
rs372806706	PTK2	8	Dominant	0.016	12.79	4.887	33.47	2.08E − 07	0.0001061	0.0006368			
rs372806706	PTK2	8	Additive	0.016	12.79	4.887	33.47	2.08E − 07	0.0001063	0.0006377			
rs369382658	PTK2	8	Dominant	0.016	13.62	4.463	41.58	4.49E − 06	0.001205	0.01376			
rs369382658	PTK2	8	Additive	0.016	13.62	4.463	41.58	4.49E − 06	0.001206	0.01378			
rs11285127	TDRD1	10	Dominant	0.344	5.526	2.634	11.59	6.11E − 06	0.001337	0.01871			
rs11285127	TDRD1	10	Additive	0.344	5.526	2.634	11.59	6.11E − 06	0.001338	0.01874			
rs142205645	TCL1A	14	Dominant	0.345	7.602	3.582	16.13	1.27E − 07	8.74E − 05	0.000389			
rs142205645	TCL1A	14	Additive	0.345	7.602	3.582	16.13	1.27E − 07	8.75E − 05	0.0003895			
rs141204613	TP53	17	Dominant	0.003	19.19	5.475	67.27	3.90E − 06	0.001195	0.01195			
rs141204613	TP53	17	Additive	0.003	19.14	5.477	66.85	3.75E − 06	0.001151	0.01151			
rs558351915	STAT3	17	Dominant	0.054	7.037	3.118	15.88	2.62E − 06	0.0009179	0.008021			
rs558351915	STAT3	17	Additive	0.054	7.037	3.118	15.88	2.62E − 06	0.0009191	0.008031			
rs779456792	STAT5A	17	Dominant	0.105	5.625	2.685	11.79	4.72E − 06	0.001205	0.01446			
rs779456792	STAT5A	17	Additive	0.105	5.625	2.685	11.79	4.72E − 06	0.001206	0.01447			
rs548702753	IL12RB1	19	Dominant	0.592	8.003	3.688	17.37	1.43E − 07	8.74E − 05	0.0004371			
rs548702753	IL12RB1	19	Additive	0.592	8.003	3.688	17.37	1.43E − 07	8.75E − 05	0.0004375			
rs769771815	NF2	22	Dominant	0.189	5.101	2.434	10.69	1.59E − 05	0.003255	0.04882			
rs769771815	NF2	22	Additive	0.189	5.101	2.434	10.69	1.59E − 05	0.003259	0.04889			

SNP single-nucleotide polymorphism, MAF minor allele frequency, HWE Hardy–Weinberg equilibrium, OR odds ratio, 95% CI 95% confidence interval, P P value calculated by unconditional logistic regression analysis, P1 P value FDR-calculated, P2 P value after Bonferroni correction

**Fig. 2:**
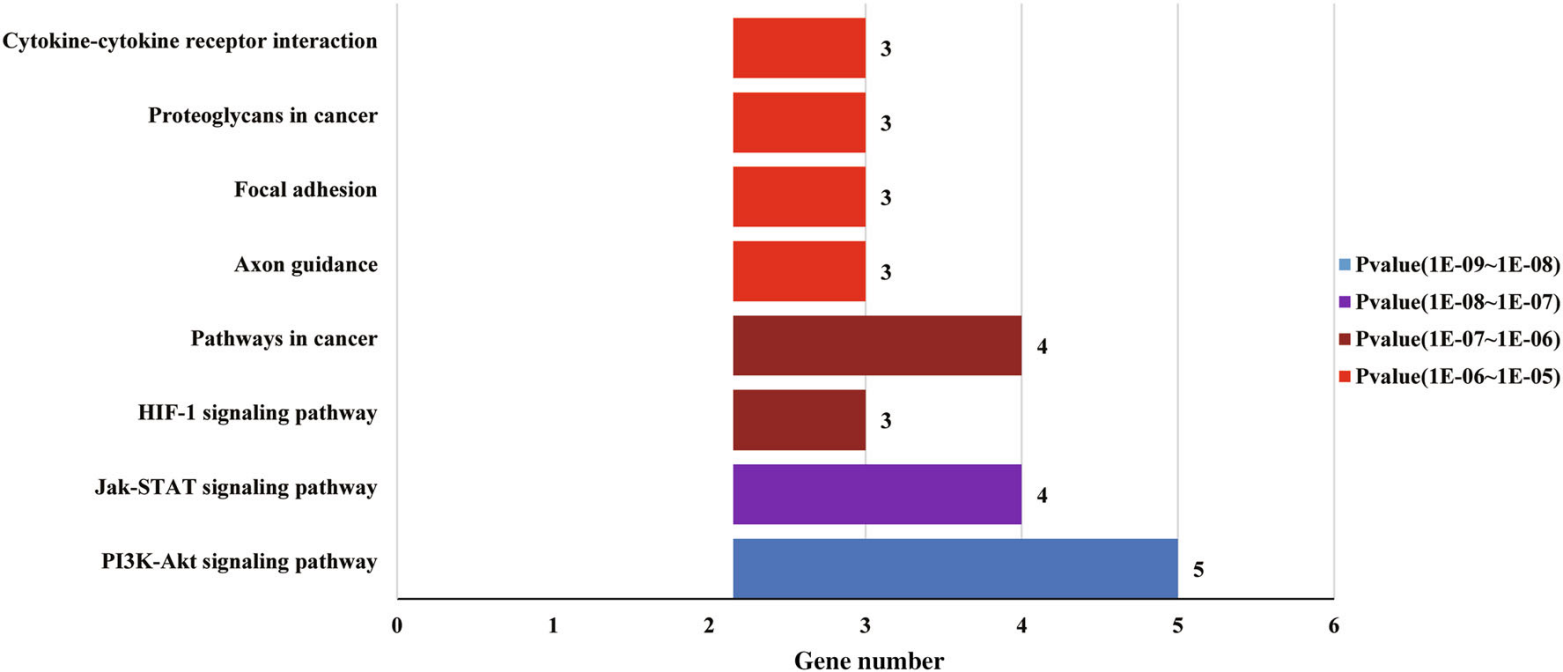
KEGG pathway analysis of differentially expressed genes. The Y-axis is the name of the KEGG metabolic pathway, and the X-axis is the number of genes annotated to the pathway (according to Table 2, *p*-value< 6.36 E − 05)

**Fig. 3:**
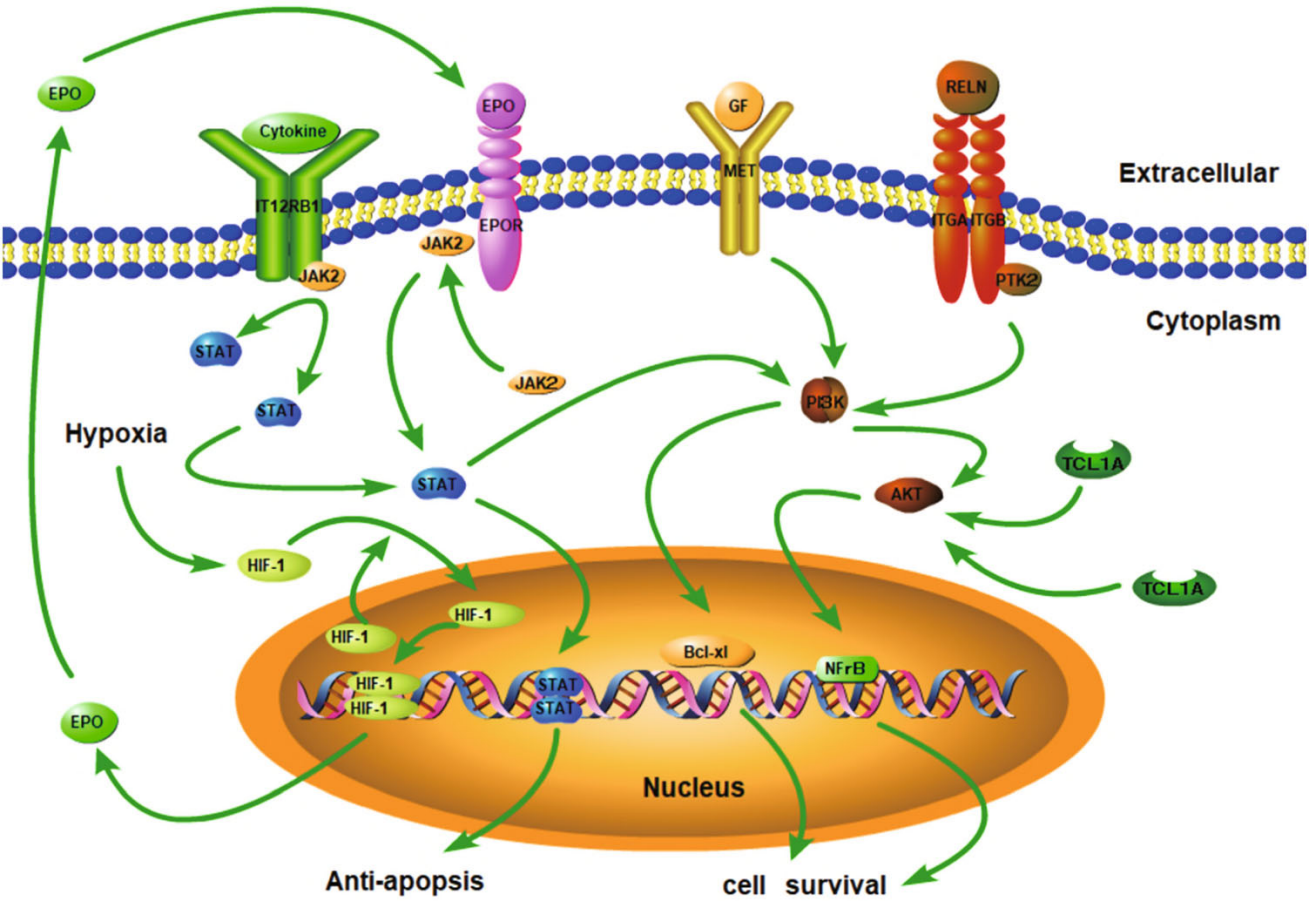
Schematic diagrams of the JAK-STAT pathway, HIF-1 pathway, and PI3K-AKT pathway. The HIF-1 signaling pathway is activated, and EPO secretion increases under hypoxic conditions. EPO then binds to the EPO receptor (EPOR). The JAK-STAT signaling pathway can also be activated and plays an anti-apoptotic role. The PI3K-Akt signaling pathway could promote the expression of the anti-apoptotic gene Bcl-xL and affect cell proliferation and differentiation. TCL1A activates the AKT pathway, which plays a role in cell survival by affecting the transcriptional activity of nuclear factor-kB (NFkB)

We adopted KEGG signaling pathways to determine gene-specific differences, and Table 3 and Fig. 2 display the results. Twelve genes were enriched in 16 signal pathways, including the PI3K-AKT pathway, JAK-STAT pathway, HIF-1 pathway, pathways in cancer, axon guidance, focal adhesion, proteoglycans in cancer, cytokine-cytokine receptor interaction, et al. Enrichment pathway information is shown in Figs. 3 (according to Table 2, *p*-value < 6.36 E − 05) and 2.

**Table 3:** KEGG pathway analysis of differentially expressed genes

Pathway	KEGG	Input	Background	*p*-value	Genes
	ID	number	number		
PI3K-Akt signaling pathway	hsa04151	5	342	3.70E − 08	PTK2, MET, TCL1A
					RELN, EPO
Jak-STAT signaling pathway	hsa04630	4	158	1.28E − 07	IL12RB1, STAT5A,
					STAT3, EPO
HIF-1 signaling pathway	hsa04066	3	103	3.98E − 06	PDK1, STAT3, EPO
Pathways in cancer	hsa05200	4	397	4.73E − 06	PTK2, STAT5A,
					MET, STAT3
Axon guidance	hsa04360	3	176	1.91E − 05	PDK1, MET, PTK2
Focal adhesion	hsa04510	3	203	2.91E − 05	RELN, MET, PTK2
Proteoglycans in cancer	hsa05205	3	205	3.00E − 05	PTK2, MET, STAT3
Cytokine-cytokine receptor interaction	hsa04060	3	265	6.36E − 05	IL12RB1, MET, EPO
Inflammatory bowel disease (IBD)	hsa05321	2	66	1.880E − 04	IL12RB1, STAT3
Central carbon metabolism in cancer	hsa05230	2	67	1.936E − 04	STAT5A, IL12RB1
Prolactin signaling pathway	hsa04917	2	72	2.227E − 04	STAT5A, STAT3
Bacterial invasion of epithelial cells	hsa05100	2	78	2.603E − 04	MET, PTK2
ErbB signaling pathway	hsa04012	2	88	3.293E − 04	STAT5A, PTK2
AGE-RAGE signaling pathway in diabetic complications	hsa04933	2	101	4.310E − 04	STAT5A, STAT3

The above results were analyzed using DAVID and KOBAS online analysis tools

PI3K-Akt: Phosphatidylinositol 3 kinase(PI3K) /protein kinase B(AKT), Jak-STAT: Janus kinase (JAK)/ signal transducer and activator of transcription (STAT), HIF-1: Hypoxia inducible factor-1 (HAF-1), ErbB: Receptor tyrosine-protein kinase erbB-2, i.e. Her2, human epidermal growth factor receptor 2 (HER2), AGE-RAGE: advanced glycation end products (AGE) / receptor for dvanced glycation end products (RAGE)

## Discussion

### Genomics and Bioinformatics Enhance the Study of HAPC

Upon completing the Human Genome Project, we will have a more comprehensive understanding of population-specific genomic variations and the interactions between genes and environmental factors. This information will help us quickly determine mutation sites related to diseases to use genetic information to establish the relationship between sequence variation and disease risk and help prevent, diagnose, and treat diseases [18, 19].

HAPC seriously intimidates the fitness of the population of the plateau. In Tibet, the increased Hb concentration enhances the efficiency of carrying oxygen to adapt to altitude hypoxia. This reaction is an imperative factor for populations, who adapt to high altitudes. We found an increased risk of HAPC associated with SNPs in *EPO*, *STAT3*, *PDK1*, *STAT5A*, *IL12RB1*, *PTK2*, *MET*, *TCL1A*, *RELN*, *RUNDC3B*, *TDRD1*, and *NF2* in the Tibetan population. We found that these genes were mostly enriched in the PI3K-AKT, JAK-STAT, and HIF-1 pathways through KEGG pathway analysis.

Notably, the genes' distributions varied widely from the different pathways based on the KEGG pathway analysis. From the perspective of the pathways, three genes *EPO*, *STAT3*, and *PDK1* were enriched in HIF-1 pathways; four genes, *EPO*, *STAT3*, *STAT5A*, and *IL12RB1*, were enriched in JAK-STAT pathways; five genes *EPO*, *PTK2*, *MET*, *TCL1A*, and *RELN* were enriched in PI3K-AKT pathways. Likewise, *EPO* gene was enriched in these three pathways; *STAT3* gene was enriched in two pathways; excluding *RUNDC3B*, *TDRD1*, and *NF2* genes, other genes were positioned in one pathway.

### The Impact of Genes Enriched in the HIF-1 Pathway for Erythrocytes

Hypoxia-inducible factor one (HIF-1), as a transcription factor, is composed of two subunits, which include an induced expressed part of HIF-1α subunit and a control structure of HIF-1β subunit. Without hypoxia, HIF-1α subunit would never be ubiquitinated and hydrolyzed by proteasomes easily [20]. Meanwhile, complex regulation of signal transduction cascades, which are mediated by cytokines and their homologous receptors, may affect the formation of HAPC. When the body is in a low-oxygen environment, its red blood cells increase. The HIF-1 signaling pathway may activate this.

*EPO* is the earliest hypoxic adaptation gene, and its polymorphism is related to the formation of HAPC [21]. Our results conformed these findings as we found that the rs773485910 of the *EPO* gene was associated with an increased prevalence of HAPC. EPO, endogenous glycoprotein hormone, has a molecular weight of 39 KD, with 166 residues. It is a member of the hematopoietic cytokine family and is primarily involved in erythropoiesis. EPO is principally secreted by liver cells in infancy and by kidney cells in adulthood. When the body immerses in a low-oxygen environment, the acetylation level of HIF-1 alpha increase, and there is enhanced transcriptional activation of a series of target genes, stimulating increased secretion of EPO by the kidneys [22]. Via binding to the EPO receptor (EPOR) on red cell progenitors in the bone marrow, circulating EPO can arouse an increase in the amount of erythrocytes [21]. However, small amounts are also expressed in other vital organs, such as the brain, spleen, lungs, testicles, and placental tissue. At average oxygen concentrations, the EPO content of blood is low, and its primary role relates to the renewal of aging erythrocytes [23].

*PDKs* are serine protein kinase genes located on chromosome 2, mainly expressed in the heart, bone marrow, kidneys, and skin. There are four isomers of PDK–PDK1, PDK2, PDK3, and PDK4. PDK1, PDK2, and PDK3 are mainly involved in cellular glucose metabolism, while PDK4 is engaged primarily in cell fat metabolism. Previous studies have shown that *PDK1* and *PDK4* genes are associated with erythrocyte polymorphism in Chuvash decent people. Our research found that rs529091195 of the *PDK* gene was associated with HAPC, which might be caused by significant upregulation of HIF due to chronic hypoxia. Upregulated HIF can promote PDK1 expression, affecting the anaerobic oxidation of cells and providing energy for normal cellular life activities [24].

Moreover, by retrieving PDK1 in the KEGG pathway database, we acquire that PDK1 also brings a crucial impact in the PI3K-AKT pathway and is activated by plasma membrane intrinsic protein 3 (PIP3). Activated PDK1 fully activates adjacent protein kinase B (AKT) and regulates downstream AGC family protein kinase activity [25–27]. These actions allow PDK1 to control the physiological effects of insulin and growth factors, increase glucose uptake, promote glycogen and protein synthesis, and provide energy for cell proliferation and differentiation [28]. In this way, PDK1 influences the development of HAPC. Once activated, the PI3K-AKT pathway can also play an anti-apoptotic role, resulting in increased erythrocyte accumulation and promoting HAPC [11].

### The Impact of Genes Enriched in the JAK-STAT Pathway for Erythrocytes

Important hematological factors widely involve the JAK-STAT pathway. When EPO binds to the EPO receptor, the JAK-STAT signaling pathway can also be activated [29]. Among those principal kinases involved in mediating EPO-responsive signal transduction, the Jak2 protein tyrosine kinase was identified for the first time by the researchers. JAK2 binds to EPOR at the bottom of the cytoplasm, causing JAK2 phosphorylation, which leads to tyrosine phosphorylation and coupling of STAT-5, affecting cell proliferation and differentiation [30].

STATs consist of seven separate members, STAT1, STAT2, STAT3, STAT4, STAT5A, STAT5B, and STAT6. Furthermore, they are active in many cell signaling pathways and have essential effects on innate immunity, acquired immunity, cell proliferation, differentiation, and survival [31]. We found that rs558351915 of the *STAT3* gene and rs779456792 of the *STAT5A* gene were related to the formation of HAPC. Previous studies have shown that different STAT family members can interact in cell signaling pathways to regulate target genes' expression [32]. The JAK-STAT pathway plays an imperative role in cellular erythropoiesis, proliferation, and differentiation. STAT3 and STAT5 are essential phosphorylated kinases in the JAK-STAT cell signaling pathway. Meanwhile, EPO binds to the EPO receptor to activate and phosphorylated JAK2; and tyrosine phosphorylated and coupled with STAT5. Then, JAK1 and STAT5 upregulate the expression of membrane proteins, cytoskeleton, hematopoietic growth factor-related genes, and downstream target genes, which contain anti-apoptotic genes *Bcl-xl* and *Bcl-2*. Moreover, the PI3K-AKT pathway is a downstream effector of JAK2-STAT5 signaling. Also, STAT5 regulates and promotes hemoglobin expression, thereby induces the proliferation of red blood cells (RBC). In a word, it can increase the RBCs' count in the body's blood [33].

### The Impact of Genes Enriched in the PI3K-AKT Pathway for Erythrocytes

The PI3K-Akt signaling pathway is also called the "cell survival signaling pathway," which can protect cells from inactivation and affect cell proliferation and differentiation by being activated. This signaling pathway brings a substantial impact on the process of erythropoiesis and could downregulate apoptosis by regulating apoptosis-related molecules. This process promotes the expression of the anti-apoptotic gene *Bcl-xL* and plays a critical anti-apoptotic role [34]. In particular, PI3K-AKT pathways affect HIF-1α transcriptional activity in hypoxia [35, 36], sequentially transactivating EPO, eventually resulting in erythrocytosis. Therefore, the PI3K-AKT signal pathway appeared to be involved in the mechanism of decreased erythroblasts apoptosis.

*PTK2* genes are enriched in the PI3K-AKT signaling pathways. Upon cell-extracellular matrix (cell-ECM) contact, PTK2 can be recruited into focal plaques and is rapidly autophosphorylated to recruit other scaffolds and signal molecules to activate the downstream PI3K-AKT signaling pathways [37]. Studies also indicated that HIF-1 gene knockout could effectively inhibit the accumulation of HIF-1 protein and the expression of PTK2 mRNA. Meanwhile, this study also indicated that PTK2 activation was inhibited; the phosphorylation levels of downstream AKT were significantly reduced. These reductions indicated that PTK2 induced the phosphorylation of AKT. The transcriptional activation of PTK2, mediated by HIF-1, protects cells from inactivation and can affect cells' proliferation and differentiation by activating the cell survival signaling pathways and inhibiting the pro-apoptotic signaling pathways.

*MET* gene is enriched in the PI3K-AKT signaling pathways, located on chromosome 7. Its product is a sort of receptor tyrosine kinase of proteins, with about 110kB in size. MET is mainly expressed in the liver and kidneys, bone marrow cells. The mature MET protein is transmembrane, a dimer complex composed of α and β subunits. There are three structural regions, of which the intracellular domain contains the binding sites for many signal molecules MET activation of the PI3K-Akt signaling pathways can promote cell proliferation and prevent cell apoptosis [38, 39]. In addition to activating the above signaling pathways, MET can also interact with cell death receptors on cell membranes to play an anti-apoptotic role (eg. Fas, FasL). These findings illustrate that MET receptors play a direct role in preventing apoptosis.

### RUNDC3B, TDRD1 and NF2 Genes Influence Erythrocytes

RUNDC3B (RUN domain containing 3B) is located on chromosome 7 and is widely expressed in the adrenal glands, brain, liver, small intestines, and other tissues. Although the biological function of RUNDC3B has not been determined, decreased expression of RUNDC3B may result in lymphoid malignancies [40]. In our study, we indicate that the rs527802276 of the RUNDC3B gene was associated with HAPC. Studies have shown that RUNDC3B and RUNDC3A (a Rap2-interacting protein) have high homology, and RUNDC3B also has a similar integration effect like RUNDC3A. The Rap protein family constitutes a subgroup of the Ras superfamily and works as a molecular "switch" regulating various cell functions, such as proliferation, differentiation, and other cell activities [41]. RUNDC3B contains a RUN domain in its N-terminal region, which is an essential component of the mitogen-activated protein kinase (MAPK) cascade. Furthermore, Rap2 interacts with MAP4K4 through its C-terminal citron homology domain. MAP4K4 is a kind of the STE20 protein kinases and regulates c-Jun N-terminal kinase (JNK). Rap2 enhances MAP4K4-induced activation of JNK. So, RUNDC3B appears to play an essential role in activating c-JUN and c-Fos transcription factors, leading to the expression of c-JUN and c-Fos in the nucleus. Its product AP1 can bind to DNA sequences and induce cell proliferation and differentiation.

The *TDRD1* gene on chromosome 10 is mainly expressed in the prostate and testes, and a small amount is expressed in the kidneys. Studies have shown that *TDRD1* is involved in spermatogenesis. The mutation of the *TDRD1* gene is related to spermatogenesis disorder in Han males [42]. In our study, rs11285127 of the *TDRD1* gene was involved in the development of HAPC.

NF2 is located on chromosome 22, and its coding product is Merlin. It has a protein with a similar structure to ERM family members. Mutation of the gene is associated with neurofibromatosis type II [43]. In our study, the rs779456792 locus of the NF2 gene was associated with HAPC. The Merlin protein may regulate the PI3K-Akt signaling pathway by interacting with PI3K and other molecules. In this way, the Merlin protein controls cell survival, proliferation, differentiation, and development of RBC, thereby affecting the formation of RBCs [44].

## Conclusion

*PDK1* (rs529091195), *RUNDC3B* (rs527802276), *EPO* (rs773485910), *RELN* (rs397889442), *MET* (rs551879100), *TDRD1* (rs11285127), *PTK2* (rs372806706, rs369382658), *TCL1A* (rs142205645), *STAT3* (rs558351915), *STAT5A* (rs779456792), *IL12RB1* (rs548702753) and *NF2* (rs779456792) genes are associated with increased risk of HAPC. Through KEGG pathway analysis, we found that these genes were mostly enriched in the PI3K-AKT, JAK-STAT, and HIF-1 pathways.

We are hopeful that our results will provide a reference for the etiology research of HAPC. However, additional genetic risk factors and functional investigations are necessary to confirm our results further.

## Supplementary Information

Below is the link to the electronic supplementary material.

Supplementary file1 (DOCX 23 kb)

Supplementary file2 (DOCX 15 kb)
