# Supplementary material for: Targeted Sequencing Identifies the Genetic Variants Associated with High-altitude Polycythemia in the Tibetan Population
Source: Indian J Hematol Blood Transfus. 2021 Aug 3;38(3):556–65. doi: 10.1007/s12288-021-01474-1 (PMC9209555; doi:10.1007/s12288-021-01474-1)
Supplement: Supplementary file 1 — Supplementary file1 (DOCX 23 kb) [file 12288_2021_1474_MOESM1_ESM.docx]

Supplementary materials 1. The list of sequenced genes

| ABCB1 | CHRM1 | ENO2 | GNGT2 | IGF1R | IL9R | LEP | NFKB1 | PIK3CD | RPS6 | TLR2 |
| --- | --- | --- | --- | --- | --- | --- | --- | --- | --- | --- |
| ABCG2 | CHRM2 | ENO3 | GP1BA | IGF2 | INPP5F | LEPR | NGF | PIK3CG | RPTOR | TLR4 |
| ACOX1 | CHUK | EP300 | GPI | IKBKB | INS | LEPROT | NGFR | PIK3R1 | RTN4R | TNC |
| ADAM17 | CISH | EPAS1 | GRB2 | IKBKG | INSR | LIF | NLK | PIK3R2 | RTN4RL1 | TNFRSF18 |
| AGT | CLCF1 | EPHA2 | GSK3B | IL10 | IRF9 | LIFR | NMI | PIK3R3 | RTN4RL2 | TNFRSF1A |
| AKT1 | CNTF | EPO | GSTP1 | IL11 | IRS1 | LOX | NOS1 | PIK3R5 | RWDD3 | TNFSF18 |
| ALDOA | COL1A1 | ERBB2 | GYS1 | IL11RA | IRS2 | LPAR1 | NOS2 | PIM1 | RXRA | TP53 |
| ANGPT1 | COL2A1 | ERBB4 | HCLS1 | IL12A | ITGA1 | LPAR2 | NOS3 | PIWIL2 | SERPINE1 | TRRAP |
| ANGPT2 | COL4A3 | F2R | HES1 | IL12B | ITGA10 | LPAR3 | NOTCH1 | PKD1 | SGK1 | TSC1 |
| ANGPT4 | COL6A3 | FASLG | HES5 | IL12RB1 | ITGA11 | LPAR4 | NOX1 | PKD2 | SGK2 | TSC2 |
| ARL2BP | COPS5 | FER | HGF | IL12RB2 | ITGA2 | LPAR5 | NOX3 | PKM | SGK3 | TSLP |
| ARNT | CREB1 | FGF1 | HGS | IL13 | ITGA2B | LPAR6 | NPPA | PKN1 | SH2B2 | TWIST1 |
| ASPH | CREB3 | FGF2 | HIF1A | IL13RA1 | ITGA3 | LRRC15 | NR1I2 | PLCG1 | SLC2A1 | TYK2 |
| ASPN | CREB5 | FGFR1 | HK1 | IL13RA2 | ITGA4 | LRRC19 | NR4A1 | PLCG2 | SLC6A8 | UROD |
| ASZ1 | CREBBP | FGFR2 | HK2 | IL15 | ITGA5 | LRRC4 | NRAS | PLCXD1 | SOCS1 | VEGFA |
| ATF2 | CRLF1 | FGFR3 | HK3 | IL15RA | ITGA6 | LRRC4B | NT5E | PLCXD1 | SOCS2 | VEGFB |
| ATF4 | CRLF2 | FGFR4 | HKDC1 | IL17D | ITGA7 | LRRC4C | NYX | PLCXD2 | SOCS3 | VEGFC |
| ATF6B | CRLF2 | FHL1 | HMOX1 | IL18 | ITGA8 | LRRC66 | OSM | PLCXD3 | SOCS4 | VHL |
| BAD | CRLF3 | FIGF | HRAS | IL19 | ITGA9 | LRRTM1 | OSMR | PODN | SOCS5 | VHLL |
| BCL2 | CRTC2 | FLRT1 | HSP90AA1 | IL2 | ITGAV | LRRTM3 | P4HB | PODNL1 | SOCS6 | VTN |
| BCL2L1 | CSF1 | FLRT2 | HSP90AB1 | IL20 | ITGB1 | LRRTM4 | P4HTM | PPP2CA | SOCS7 | VWF |
| BCL2L11 | CSF1R | FLRT3 | IBSP | IL20RA | ITGB3 | LRTM1 | PCK2 | PPP2R1A | SOX2 | XYLT2 |
| BGN | CSF2 | FLT1 | ID2 | IL20RB | ITGB4 | LTBR | PDGFA | PRKAA1 | SPP1 | YWHAB |
| BRCA1 | CSF2RB | FLT3 | IFI35 | IL21 | ITGB5 | LYN | PDGFB | PRKAA2 | STAM | YWHAE |
| CA12 | CSF3 | FLT4 | IFNA1 | IL21R | ITGB6 | MAP1LC3B | PDGFC | PRKCA | STAMBP | YWHAG |
| CA9 | CSH1 | FN1 | IFNA10 | IL22 | ITGB7 | MAP2K1 | PDGFD | PRKCB | STAT1 | YWHAH |
| CAMK2A | CTF1 | FOXO3 | IFNA14 | IL23A | ITGB8 | MAP2K2 | PDGFRA | PRKCD | STAT2 | YWHAQ |
| CAMK2B | CTR9 | G6PC | IFNA16 | IL23R | JAK1 | MAPK1 | PDGFRB | PRKCG | STAT3 | YWHAZ |
| CAMK2D | CUL2 | GABRP | IFNA17 | IL24 | JAK2 | MAPK3 | PDHA1 | PRKCQ | STAT4 | ZEB1 |
| CAMK2G | CXCL12 | GAPDH | IFNA2 | IL26 | JAK3 | MCL1 | PDHA2 | PRL | STAT5A |  |
| CASP9 | CXCR4 | GFAP | IFNA21 | IL27 | JUN | MDM2 | PDHB | PRLR | STAT5B |  |
| CAV1 | CYBB | GH1 | IFNA4 | IL27RA | KDM5B | MET | PDK1 | PSMD9 | STAT6 |  |
| CCBL2 | CYP1B1 | GH2 | IFNA5 | IL2RA | KDR | MKNK1 | PDK3 | PTEN | STK11 |  |
| CCL2 | DCN | GHR | IFNA6 | IL2RB | KIT | MKNK2 | PDPK1 | PTK2 | SYK |  |
| CCL5 | DDIT4 | GNB1 | IFNA7 | IL2RG | KITLG | MLST8 | PECAM1 | PTK2B | TBC1D3K |  |
| CCND1 | DDX4 | GNB2 | IFNA8 | IL3 | KLF4 | MMP14 | PFKFB3 | PTK6 | TCEB1 |  |
| CCND2 | DOT1L | GNB3 | IFNAR1 | IL31RA | KRAS | MMP2 | PFKL | PTPN1 | TCEB2 |  |
| CCND3 | EDN1 | GNB4 | IFNAR2 | IL3RA | LAMA1 | MOV10L1 | PGF | PTPN11 | TCL1A |  |
| CCNE1 | EFNA1 | GNB5 | IFNB1 | IL3RA | LAMA2 | MPL | PGK1 | PTPN2 | TCL1B |  |
| CCR2 | EGF | GNG10 | IFNE | IL4 | LAMA3 | MSH2 | PHF11 | PTPN6 | TDRD1 |  |
| CD19 | EGFR | GNG11 | IFNG | IL4R | LAMA4 | MSH6 | PHLPP1 | RAC1 | TDRD5 |  |
| CD300A | EGLN1 | GNG12 | IFNGR1 | IL5 | LAMA5 | MST1 | PIAS1 | RAF1 | TDRKH |  |
| CD40 | EGLN2 | GNG13 | IFNGR2 | IL5RA | LAMB1 | MT1X | PIAS2 | RBL2 | TEK |  |
| CDC37 | EGLN3 | GNG2 | IFNK | IL6 | LAMB2 | MT2A | PIAS3 | RBM43 | TERT |  |
| CDK2 | EIF4B | GNG3 | IFNL1 | IL6R | LAMB3 | MTCP1 | PIAS4 | RBX1 | TF |  |
| CDK4 | EIF4E | GNG4 | IFNL3 | IL6ST | LAMB4 | MTOR | PIBF1 | RELA | TFRC |  |
| CDK6 | EIF4E1B | GNG5 | IFNL4 | IL7 | LAMC1 | MYB | PIGU | RELN | THBS1 |  |
| CDKN1A | EIF4E2 | GNG7 | IFNLR1 | IL7R | LAMC2 | MYC | PIK3AP1 | RHEB | THEM4 |  |
| CDKN1B | EIF4EBP1 | GNG8 | IFNW1 | IL9 | LAMC3 | NANOG | PIK3CA | RNF41 | THPO |  |
| CHAD | ENO1 | GNGT1 | IGF1 | IL9R | LDHA | NF2 | PIK3CB | RORC | TIMP1 |  |
