# Supplementary material for: Targeted Sequencing Identifies the Genetic Variants Associated with High-altitude Polycythemia in the Tibetan Population
Source: Indian J Hematol Blood Transfus. 2021 Aug 3;38(3):556–65. doi: 10.1007/s12288-021-01474-1 (PMC9209555; doi:10.1007/s12288-021-01474-1)
Supplement: Supplementary file 2 — Supplementary file2 (DOCX 15 kb) [file 12288_2021_1474_MOESM2_ESM.docx]

Table2

Our results revealed that rs529091195 (OR = 7.624, 95% CI = 3.585–16.21, Dominant P=1.314e-07, P1=8.742e-05, P2=4.026e-04; Additive P=1.314e-07, P1=8.75e-05, P2=4.031e-04); rs527802276 (OR=12.320, 95% CI=4.933–30.770, Dominant P=7.575e-08, P1=8.742e-05, P2=2.320e-04, Additive P=7.575e-08，P1=8.750e-05，P2=2.323e-04）； rs773485910 (OR =4.657, 95% CI = 2.278–9.519 , Dominant P=2.471e-05, P1=4.731e-03, P2=0.076; Additive P=2.471e-05, P1=4.737e-03, P2=0.076); rs397889442 (OR = 4.282, 95% CI=2.050–8.944, Dominant P=1.087e-04, P1=0.0196, P2=0.333; Additive P=1.087e-04, P1=0.020, P2=0.333); rs551879100 (OR= 6.679, 95% CI=2.950–15.120，Dominant P=5.259e-06, P1=1.239e-03，P2=0.016, Additive P=5.259e-06, P1=0.001, P2=0.016); rs372806706 (OR=12.790, 95% CI=4.887–33.470, Dominant P=2.079e-07, P1=1.061e-04, P2=6.368e-04, Additive P=2.079e-07, P1=1.063e-04, P2=6.377e-04); rs369382658 (OR=13.620, 95% CI=4.463–41.580, Dominant P=4.493e-06, P1=1.205e-03, P2=0.014，Additive P=4.493e-06, P1=1.206e-03, P2=0.014); rs11285127 (OR=5.526, 95% CI=2.634–11.590, Dominant P=6.109e-06, P1=1.337e-04, P2=0.019; Additive P=6.109e-06, P1=1.338e-03, P2=0.019); rs142205645 (OR=7.602, 95% CI=3.582–16.13, Dominant P=1.27e-07, P1=8.742e-05, P2=3.890e-04; Additive P=3.900e-06, P1=1.195e-03, P2=0.012); rs558351915 (OR=7.037, 95% CI=3.118–15.880, Dominant P=2.619e-06, P1=9.179e-04, P2=8.021e-03; Additive P=2.619e-06, P1=9.191e-04, P2=8.031e-03); rs779456792 (OR=5.625, 95%CI=2.685–11.790, Dominant P=4.719e-06, P1=1.205e-03, P2=0.014, Additive P=4.719e-06, P1=1.206e-03, P2=0.014); rs548702753 (OR = 8.003, 95% CI = 3.688–17.37, Dominant P=1.427e-07, P1=8.742e-05, P2=4.371e-04; Additive P=1.426e-07, P1=8.75e-05, P2=4.375e-04); rs769771815 (OR=5.101, 95% CI=2.434–10.690, Dominant P=1.594e-05, P1=3.255e-03, P2=0.049, Additive P=1.594e-05, P1=3.259e-03, P2=0.049).
